# Communication Support for People with ALS

**DOI:** 10.1155/2011/714693

**Published:** 2011-04-14

**Authors:** David Beukelman, Susan Fager, Amy Nordness

**Affiliations:** Institute for Rehabilitation Science and Engineering Madonna Rehabilitation Hospital and University of Nebraska, 202 Barkley Memorial Center, P.O. Box 830732, Lincoln, NE 68583-0732, USA

## Abstract

Almost all people with amyotrophic lateral sclerosis (ALS) experience a motor speech disorder, such as dysarthria, as the disease progresses. At some point, 80 to 95% of people with ALS are unable to meet their daily communication needs using natural speech. Unfortunately, once intelligibility begins to decrease, speech performance often deteriorates so rapidly that there is little time to implement an appropriate augmentative and alternative communication (AAC) intervention; therefore, appropriate timing of referral for AAC assessment and intervention continues to be a most important clinical decision-making issue. AAC acceptance and use have increased considerably during the past decade. Many people use AAC until within a few weeks of their deaths.

## 1. Introduction

Almost all people with amyotrophic lateral sclerosis (ALS) experience a motor speech disorder as the disease progresses. Initial symptoms typically do not interfere with speech intelligibility and may be limited to a reduction in speaking rate, a change in phonatory (voice) quality, or imprecise articulation. At some point in the disease progression, 80 to 95% of people with ALS are unable to meet their daily communication needs using natural speech. In time, most become unable to speak at all [1]. For them, communication support involves a range of augmentative and alternative communication (AAC) strategies involving low- and high-technology (speech generating device) options [2]. Clinical decision-making related to communication is quite complex as screening, referral, assessment, acquisition of technology, and training must occur in a timely manner, so when residual speech is no longer effective, AAC strategies are in place to support communication related to personal care, medical care, social interaction, community involvement, and perhaps employment. Although there is considerable research on the speech characteristics of people with ALS, it is the primary purpose of this paper to review the published research related to communication supports for people with ALS whose natural speech no longer meets their communication needs.

## 2. Speech Characteristics

 ALS involves both upper and lower motor neurons; therefore, it results in mixed dysarthria of the flaccid-spastic type [3, 4]. In the early stages of ALS when dysarthria is mild, either spasticity or flaccidity is predominant. As ALS progresses and dysarthria becomes severe, profound weakness resulting in reduced movement of the speech musculature and severely reduced phonation become increasingly common [5, 9]. 

Changes in speech patterns or speaking rate typically occur before a decrease in speech intelligibility [6–9]. Initially, speaking rate gradually slows; however, speech intelligibility initially remains relatively high. In time, dysarthria becomes apparent to people with ALS and their listeners, and then speech intelligibility decreases such that communication effectiveness is reduced at first in adverse speaking situations, such as noisy crowds, and then in all situations. The results of a study by Ball et al. [10] revealed that perceptions of communication effectiveness for speakers with ALS were quite similar for the speakers and their frequent listeners across 10 different social situations. ALS speakers and their listeners reported a range of communication effectiveness depending upon the adversity of specific social situations.

## 3. Speech Intervention

A recent review of ALS communication research [11] concluded that, because of the pathophysiology and the degenerative nature of ALS, speech treatment strategies that are designed to increase strength or mobility of the oral musculature are not recommended. People with ALS, or those close to them, often request oral exercises to improve strength and mobility for speech and swallowing, as strengthening exercises seem intuitive to them as way to increase performance. However, such exercise programs should be discouraged, and those with ALS should be informed that the speaking that they do each day provides a sufficient amount of speech mechanism activity and exercise. 

Speech intervention should focus on learning to conserve energy for priority speaking tasks and to rest often to reduce fatigue instead of increasing effort and use with speech exercises. ALS speakers should learn to avoid adverse speaking/listening situations by muting the television, inviting people to speak with them in a quiet place rather than in a crowded room, and using voice amplification when speaking in noisy environments to reduce the effort required [1, 12]. As speech becomes difficult to understand, many ALS speakers supplement their speech by identifying the first letter of each word on an alphabet board (alphabet supplementation) or by identifying the topic on a communication board (topic supplementation). Although improvements of speech intelligibility have been documented for these supplementation procedures in a practice guideline article by Hanson et al. [13], none of this research has involved ALS speakers.

It is often difficult for speakers with ALS, their family members and medical personnel to consider AAC strategies when they are still using residual speech to meet daily communication needs. However, their speaking rate should be clinically monitored such that the referral for an AAC intervention is initiated in a timely manner. With sufficient education and preparation, people with ALS and their decision-makers are ready to examine their AAC options. However, speech deterioration can be so rapid that individuals can be left with limited communication options, if they are not prepared to act in a timely manner.

## 4. Timely Referral for Communication Support

Often people with ALS, their family members, and, at times, their medical team, do not wish to consider an AAC decision until their deteriorating speech intelligibility limits their communication effectiveness. Unfortunately, once intelligibility begins to decrease, speech performance often deteriorates so rapidly that there is little time to implement an appropriate AAC intervention. Appropriate timing of referral for AAC assessment and intervention continues to be a most important clinical decision-making issue. Yorkston et al. [8] initially suggested that speaking rate reduction precedes decreases in intelligibility in people with ALS. Ball et al. [14, 15] evaluated the speech performance of 158 different people at 3-month intervals from diagnosis to death. These authors reported that speaking rate is a relatively good predictor of intelligibility deterioration for patients with spinal, bulbar, or mixed ALS. They recommend that ALS patients be referred for AAC assessment when their speaking rates reach 125 words per minute on the Speech Intelligibility Test (Sentence Subtest) [16]. The mean speaking rate on this test for adults without disability is 190 words per minute. This computerized test supports the efficient measurement of speaking rate in clinical settings, so speaking rate information can be shared with the patient and family immediately during clinical visits. This helps patients and their families monitor changes over time, prepare for an AAC evaluation, and it reinforces their understanding of rate and intelligibility. Using the Speech Intelligibility Test (Sentence Subtest), speaking rate can be accurately monitored over the telephone if a patient lives at a distance or is unable to travel due to illness weather, or support issues [17]. It should be noted that speech intelligibility could not be objectively assessed over the telephone, as a clinical measure of understandability.

Nordness et al. [18] reviewed the records of nearly 300 people with ALS served by 3 different AAC centers. Each of these centers implemented the referral guideline of 125 words per minute on the Speech Intelligibility Test (Sentence Subtest). The authors reported that 88% of the people in the sample received timely AAC assessments. “Of the 12% who received “late” referrals, most (93%) were delayed because of a late referral by their physician, travel demands, and other interfering health conditions, while a few (7%) received a delayed assessment because of factors related to the person with ALS or caregivers.” Most physicians who did not refer in a timely manner were general practitioners, neurologists not associated with a multidisciplinary neuromuscular clinic, or medical staff of long-term care facilities. A higher percentage of females than males were identified as receiving late AAC assessments.

## 5. AAC Acceptance

Ball et al. [6] reported that approximately 95% of people with ALS in the Nebraska ALS Database become unable to speak at some point prior to death. AAC acceptance and use have increased considerably during the past decade. Prior to 1996, approximately 72% of men and 74% of women for whom AAC technology was recommended accepted and used the technology [19]. However, in a more recent report by Ball et al. [6], 96% of people with ALS for whom speaking rate was monitored and AAC assessment was recommended in a timely manner accepted and used AAC, with 6% delaying but eventually accepting the technology. No differences were reported for males and females. In the review by Ball et al. [10] those who rejected AAC reported a cooccurring functional dementia or experienced multiple severe health issues, such as cancer, in addition to ALS.

AAC acceptance involves the patient with ALS as well as family members and other caregivers. Richter et al. [20] investigated attitudes toward AAC options by people with ALS, caregivers, and unfamiliar listeners. The results indicated agreement among these groups with a strong preference for AAC use for being over difficult to understand speech or a low-tech communication book. Fried-Oken et al. [21] surveyed AAC caregivers. They reported very positive attitudes toward AAC technology. Those with greater AAC technology skills reported greater rewards associated with caregiving. They reported increased perception of social closeness to the individual with ALS and less difficulty in providing care.

## 6. AAC Use

People with ALS use AAC technology for an extended period of time. Mathy et al. [19] reported on 33 people with ALS between 1988 and 1996 and found that the mean duration of use was 14 months. More recent data from the Nebraska ALS Database have revealed that people with ALS use their AAC technology with an average of 24.9 months for those with bulbar ALS and 31.1 months for those with spinal ALS. Many people used AAC until within a few weeks of their deaths. Because 15% of the participants in this study continued to use their AAC technology at the time the report was completed and were supported by invasive ventilation, the mean duration of use reported likely underestimated the length of use for this sample and for people with ALS in general [1].

Due to the extended use of AAC with deteriorating levels of physical control, it is imperative that recommended technology has adjustable access options to meet the range of motor capability as the disease progresses, that is, people with ALS should be fitted with AAC technology that supports multiple access methods such as allowing them to transition from hand access to scanning and/or head/eye-tracking. Many AAC devices now incorporate a variety of access options so that the technology can continue to meet the needs of the user despite a decline in physical capability. The sensitivity of dynamic touch screens can be adjusted to allow for lighter touch. The improved sensitivity of head-tracking technology has allowed many to use this access method with minimal head/neck movement control. 

Perhaps the most significant advancement in access technology has occurred with the widespread availability of eye-tracking systems to allow cursor control with eye movement to access high-technology AAC devices. As the disease progresses, many ALS patients require the use of eye-tracking for several reasons. First, eye-tracking is often the least fatiguing movement for AAC access. Eye gaze is natural, and eye muscles generally do not fatigue with use [22, 23]. Compared to other access methods such as switch-activated scanning, eye-tracking is often reported to be the least fatiguing access method by people with ALS [24]. Others have reported that eye-tracking technology requires relatively little effort [25, 26]. Second, eye gaze may be the only volitional movement that the individual continues to exhibit over time, particularly in cases where invasive ventilation has been chosen [27]. 

In a follow-up investigation of 15 people with ALS, Ball et al. [27] examined the acceptance, training, and extended use patterns of eye-tracking technology to support communication. Ninety-three percent of the participants reported successful implementation of the technology. For 53% of the participants, eye-tracking technology was selected because eye movement was the only viable access option available. The one individual who was not able to successfully use eye-tracking technology had difficulty with eyelid control, which has been noted as a potential issue in ALS [28]. 

The communicative functions served by eye-tracking devices in Ball and colleagues' [27] investigation were extensive. All of the participants (100%) used their eye-tracking device to support face-to-face communication. Other functions included group communication (43%), phone (71%), email (79%), and internet (86%). Six of the participants (43%) also reported using the eye-tracking technology to support other computer-based functions (e.g., word processing, vocation-related software programs). Others have also reported a wide range of communicative functions served by AAC for people with ALS [21, 29–31] including word processing, providing accounting services, or consulting over the phone or Internet.

## 7. Communication and Life Expectancy

Life expectancy of patients with ALS varies depending on a number of factors. Those who experience initial spinal symptoms survive approximately five times longer than those with initial bulbar (brainstem) symptoms. Life expectancy is longer for those who opt for noninvasive and invasive ventilation than for those who do not [32]. According to a database review [33], the decision to use invasive ventilation extends the length of AAC use overall, as well as the duration of time during which AAC technology must be controlled with minimal or no limb or head movement. Adequate nutrition at the time of diagnosis and artificial nutrition, such as a percutaneous endoscopic gastrostomy (PEG), as the disease progresses improves the quality of life and may extend the length of life somewhat [32, 34, 35]. It potentially could have an impact on AAC use, in that people with ALS who use artificial nutrition spend less time eating, have more energy, and have more time to participate in the social activities of their choice. Often, such participation in social situations increases the need and opportunity for AAC use.

## 8. AAC Training and Support

Training and support are an essential component of AAC service delivery for people with ALS. The significant changes in movement capabilities require that service providers not only be proactive in their AAC technology recommendations by providing technology options that can meet the changing physical needs over time, but also provide adequate training and support to ensure that the people with ALS and their caregivers can successfully implement these access strategies over time. Reports of low AAC use often accompany descriptions of minimal training or follow-up [36]. New advances in AAC technology (e.g., eye-tracking) may require a greater amount of training and intervention than other access options. Ball and colleagues [27] found that implementation of eye-tracking systems often required trouble-shooting in the form of physical or environmental compensations for successful use of the technology. For example, the use of glasses often required adjustments to the LED camera angle to separate the glint on the pupil from the glare on the glasses. Others required environmental lighting changes (e.g., changing incandescent bulbs to fluorescent lighting, dimming lights, and closing shades). The mean length of instruction provided for these people was 5 hours (range of 2–20 hours) with a mean troubleshooting time of 2.27 hours (range of 0–10 hours). 

While AAC specialists are professionals who provide the AAC intervention services such as assessment and initial instruction, AAC facilitators for people with ALS tend to be family members who typically provide ongoing support including instruction of new communication partners and caregivers, programming new messages into the AAC device, maintaining the AAC system, and interacting with the technology manufacturer if necessary [37]. Ball et al. [38] surveyed 68 people with ALS who used AAC technology. All identified a primary AAC facilitator. Ninety-six percent of the AAC facilitators were family members, most with nontechnical backgrounds. In response to a survey, these primary facilitators preferred hands-on, detailed step-by-step instruction. They reported receiving slightly over 2 hours of instruction and reported that amount of training as appropriate.

## 9. Future Research Directions

Although not documented with published research findings, AAC service delivery models for people with ALS differ considerably. The Nebraska Database was collected from a highly integrated intervention system in which a speech language pathologist with considerable AAC expertise is a regular staff member in three regional clinics that also includes a neurologist, physical therapist, occupational therapist, registered dietitian, respiratory therapist, and social worker. This AAC interventionist provides routine speech screening and education with families. When the speaking rate threshold of 125 words per minute on the Speech Intelligibility Test (Sentence Subtest) is reached, ALS patients are referred to one of three AAC specialty programs for assessment, implementation, and follow-up. The acceptance and use data for each patient are reported back to the coordinating AAC specialist involved in the AAC clinics. This process typically provides a gradual familiarization with AAC, which reflects a process reported to increase adaptation to or acceptance of other supports, such as assisted ventilation [39]. On the other hand, the Murphy [36] article documents AAC acceptance and usage associated with a much less integrated service delivery system. The authors suggest that the organization of the service delivery system may have impacted the AAC acceptance and use data reported in this study. Research is needed to investigate the impact of AAC service delivery strategies on intervention effectiveness.

The impact of cognitive function on AAC acceptance and use needs to be investigated systematically. Ball et al. [1] note that while the prevalence of cognitive impairments in people with ALS is more common than previously thought, these impairments seem to influence AAC acceptance and use in a relatively small percentage of those with ALS. As was reported earlier in this paper, a limited number of AAC patients with severe frontotemporal dementia rejected AAC intervention. A recent research summary has reported that between 10–75% of ALS patients experience cognitive impairment and between 15–41% experience a fronto-temporal dementia (FTD) as measured by neuropsychological testing, although its effect on management of ALS is unknown [40]. In preparation for this paper the authors reviewed the Nebraska Database for the ALS patients served in the last 30 months (*N* = 87). According to the multidisciplinary clinical screen, 77.0% did not demonstrate cognitive impairments, 18.4% demonstrated a mild cognitive impairment, and 4.6% demonstrated a fronto-temporal dementia (FTD). Of the patients with FTD, two were not capable of using AAC, one was able to write and gesture, and one was still able to use some speech. Of the patients with a mild cognitive impairment, 62.5% (*N* = 10) were able to communicate with AAC, 25% (*N* = 4) did not yet require AAC, and 12.5% (*N* = 2) rejected AAC. Anecdotally, the authors supported numerous patients with ALS whose cognitive limitations were of concern to the ALS clinic team, but who accepted and used high- and low-technology AAC strategies successfully to meet their communication needs. Research is needed to objectively document AAC acceptance and use related primarily to cognitive impairment. Further research is also needed to clarify the level of cognitive impairment that tends to interfere with AAC intervention.

Brain computer interface (BCI) technology has generated considerable research interest for people who are physically “locked-in” such as those in the late stages of ALS. BCI research includes invasive (implantable electrodes on or in the neocortex) and noninvasive means (including electroencephalography (EEG), magnetoencephalography (MEG), fMRI, and the less expensive near-infrared spectography (NIRS)). Non-invasive methods have been utilized more extensively than invasive methods for people with disabilities (such as those with ALS) [41–43]. While those with ALS and other conditions who are in a “locked-in” physical state have motivated research in this area, very few systems have been successful with this population. It has been postulated that some forms of cognitive impairment and changes in EEG signatures in late stage ALS may contribute to the lack of success using BCI technology as the technology was introduced after the participants had become “locked-in” [41, 44]. The most successful application for communication has occurred in people at the beginning stages of the disease [45–47]. To date, no investigations have reported of the use of BCI throughout the disease progression of ALS to determine if these people would be able to maintain training and functional of the systems.

Use of AAC interventions, including speech generating devices, is recognized as the standard of care (practice) for people with speech-related functional losses associated with ALS. Considerable research has documented the need for AAC support, as well as the acceptance, use, and effectiveness of AAC strategies for people with this medical diagnosis. As in other fields, additional research is needed to develop new intervention strategies and to document their effectiveness.
